# A Short Enquiry into the Merit of Solvents, so Far as May Be Necessary to Compare Them with the Operation of Lithotomy

**Published:** 1781-10

**Authors:** 


II. *A ſhort enquiry into the merit of ſolvents, ſo far as may be neceſſary to compare them with the operation of lithotomy. By* Jere Whitaker Newman, *member of the Corporation of Surgeons, London.* 8vo. Dodſley, *London*, 1781. 43 pages, price 1s 6d.

THE

THE design of this eſſay is to give a view of the general method of treatment by internal medicines and its effects; and, in a conciſe manner, to compare the advantages and evils reſulting from a reliance on that mode of treatment, with thoſe attending the extraction by manual operation.

The work is divided into three chapters. In the firſt the author begins with vindicating his brethren from the idea of a predilection for operations, which has been ſo commonly, though erroneouſly annexed to the character of a ſurgeon. He then offers ſome few remarks on the general theory and baſis of ſolvents, and takes occaſion to relate the ſingular but well-known circumſtance attending Mrs. Stephens's medicine in the caſe which procured her the reward from parliament.

In the ſecond chapter he mentions ſome of the cauſes which have tended to render lithotomy leſs ſucceſsful than it would otherwiſe have been. The firſt of theſe is delay, the frequency and fatality of which induced Frere Jacques to exclaim, that if lithotomy, as its oppoſers aſſerted, had killed thouſands, putting off the operation until the patient's ſtrength and conſtitution were exhauſted had deſtroyed tens of thouſands. A ſecond fecond caufe fpoken of by our author is, the long-continued courfes of lixivial and other remedies, intended as folvents: a third he fuppofes to be the contaminated air of hofpitals; and a fourth an inattention to a few particulars previous to and during the operation.——The two laft caufes are treated of in his third chapter.

Speaking of the pernicious effects of folvents our author mentions the cafe of the late Mr. Garrick, who laboured for many years under a calculous complaint. Partial to medicines and eager to embrace every thing that poffeffed or promifed any efficacy, he perfifted in the ufe of many folvents; and a violent pain in the ftomach, which latterly was frequent, he generally attributed to the effects of thefe medicines, as he had not experienced it previous to their ufe. On infpecting his body after death, the organization of the kidnies appeared to be almoft wholly deftroyed; and a ftone of confiderable fize was found in the bladder, the furface of which plainly evinced that it had not been in any degree abraded.

Mr. Newman has feen two inftances, where the patients, in a diftinct benign fpecies of the fmall pox, died, though the difeafe in the neighbourhood was by no means fatal. Both were taking folvents when the fymptoms of the fmall pox came came on. A third patient, under a ſimilar courſe, was ſeized with a putrid fever, which terminated in death, notwithſtanding the liberal uſe of bark, port wine, &c.

On the ſubject of fixed air, given as a lithontriptic, our author ſhews, that its total inefficacy in ſeveral caſes, and the want of proof that a ſtone really exiſted where it was ſuppoſed to have been beneficial, prevent any thing being decided in its favour.

He very properly recommends gentleneſs in ſearching, previous to the operation. "The "puerile circumſtance—ſays he—of the in"ſtrument being heard to reſound againſt the "ſtone, by perſons at a diſtant part of a room, "will not warrant us in moving the ſound rude"ly and rapidly in the cavity of the bladder, "where there is a poſſibility of ſtriking it "againſt the internal membrane, which, beſides "increaſing the pain, might be productive of "diſagreeable conſequences."

In ſpeaking of the defects in ſome parts of the operation, he confines himſelf chiefly to the external inciſion, which every one, in the habit of attending to operations, muſt at times have obſerved to be too ſmall for the extraction of the ſtone; the utility of a large external inci-

 ſion fion in thefe cafes feems to be generally acknowledged.

In this part of his work the author hints at the mifchiefs that have been produced by the notion that quicknefs is an indifpenfable qualification in a lithotomift. " An operator, he " obferves, muft be poffeffed of a good fhare " of coolnefs and intrepidity, who would not " be affected by thirty or forty watches being " taken out in a theatre, to determine critically " the precife time in which the operation was " performing." We hoped that after what had been fo judicioufly remarked by Mr. Pott fome years ago on this fubject, the abfurd and dangerous cuftom here alluded to was univerfally exploded.

Towards the clofe of his performance, Mr. Newman endeavours to determine what is the general fuccefs of lithotomy. For this purpofe he quotes the account given by the late Mr. Chefelden of his practice in this way, and from this, added to what has fallen under his own obfervation, during his attendance at hofpitals, he concludes, that not more than one out of eleven dies in confequence of being cut for the ftone. He obferves, that " fhould " future ages poffefs a folvent, efficacious, but

" not

"not deftructive to the fyftem, lithotomy may "perhaps be fuperfeded;" but that "till then "reafon and experience point it out as the beft "and only refource."

He concludes "with advifing thofe, who "have prudent refolution enough to undergo "the operation, to do it in time, before the "irritation of the difeafe itfelf, or the deleterious "ufe of folvents, have impaired their general "health: for in a morbid ftate of the blood "and juices, no wound can heal kindly; and "the operation, however fafe and fkilfully per- "formed, by fuch means may be rendered ha- "zardous, and frequently fatal."

